# Hypoxia Enhances Proliferation of Human Adipose-Derived Stem Cells via HIF-1ɑ Activation

**DOI:** 10.1371/journal.pone.0139890

**Published:** 2015-10-14

**Authors:** Natsuko Kakudo, Naoki Morimoto, Takeshi Ogawa, Shigeru Taketani, Kenji Kusumoto

**Affiliations:** 1 Department of Plastic and Reconstructive Surgery, Kansai Medical University, Osaka, Japan; 2 Department of Biotechnology, Kyoto Institute of Technology, Kyoto, Japan; University of Illinois at Chicago, UNITED STATES

## Abstract

**Background:**

Adipose tissue-derived stem cells (ASCs) have been recently isolated from human subcutaneous adipose tissue. ASCs may be useful in regenerative medicine as an alternative to bone marrow-derived stem cells. Changes in the oxygen concentration influence physiological activities, such as stem cell proliferation. However, the effects of the oxygen concentration on ASCs remain unclear. In the present study, the effects of hypoxia on ASC proliferation were examined.

**Methods:**

Normal human adipose tissue was collected from the lower abdomen, and ASCs were prepared with collagenase treatment. The ASCs were cultured in hypoxic (1%) or normoxic (20%) conditions. Cell proliferation was investigated in the presence or absence of inhibitors of various potentially important kinases. Hypoxia inducible factor (HIF)-1α expression and MAP kinase phosphorylation in the hypoxic culture were determined with western blotting. In addition, the mRNA expression of *vascular endothelial growth factor* (*VEGF*) and *fibroblast growth factor* (*FGF*)*-2* in hypoxic or normoxic conditions were determined with real-time RT-PCR. The effects of these growth factors on ASC proliferation were investigated. Chromatin immunoprecipitation (ChIP) of the HIF–1α-binding hypoxia responsive element in *FGF–2* was performed. HIF–1α was knocked down by siRNA, and FGF–2 expression was investigated.

**Results:**

ASC proliferation was significantly enhanced in the hypoxic culture and was inhibited by ERK and Akt inhibitors. Hypoxia for 5–15 minutes stimulated the phosphorylation of ERK1/2 among MAP kinases and induced HIF–1α expression. The levels of VEGF and FGF–2 mRNA and protein in the ASCs were significantly enhanced in hypoxia, and FGF–2 increased ASC proliferation. The ChIP assay revealed an 8-fold increase in the binding of HIF–1α to *FGF–2* in hypoxia. HIF–1α knockdown by siRNA partially inhibited the FGF–2 expression of ASCs induced by hypoxia.

**Conclusion:**

ASC proliferation was enhanced by hypoxia. HIF–1α activation, FGF–2 production, and the ERK1/2 and Akt pathway were involved in this regulatory mechanism.

## Introduction

Adipose tissue was recently shown to be a source of multipotent adult stem cells, providing enriched adipose-derived stem cells (ASCs). ASCs have the potential to differentiate into bone, cartilage, tendons, nerves, and fat when cultured under lineage-specific conditions [1] [2]. Because of the convenience of isolation and extensive proliferative and differentiation capacities in vitro, ASCs are a promising source of human stem cells for regenerative medicine. To date, various cell culture methods have been developed to more efficiently obtain stem cells while minimizing the risks to donors[3] [4].

Recent studies revealed that low oxygen tension or hypoxia affects various types of stem cells, such as embryonic stem cells [5], induced pluripotent stem cells [6], and bone marrow-derived stem cells (BMCs) [7] [8] [9]. A low oxygen environment is physiologically normal not only for most mammalian embryos, but also for adult somatic stem cells [8]. In mammalian cells, the transcriptional response to oxygen deprivation is largely mediated by hypoxia inducible factor 1 (HIF–1), which gradually increases as the oxygen concentration decreases. Expression of genes such as *vascular endothelial growth factor* (*VEGF*) and *erythropoietin* is induced to stimulate angiogenesis and hematopoiesis. ASC proliferation is enhanced in hypoxia compared with normoxia [10] [8]. Secretion of VEGF and fibroblast growth factor (FGF)-2 proteins from ASCs is increased in hypoxia [11]. However, the detailed mechanisms remain unknown. The relationship between the response of ASCs to hypoxia and cell proliferation in this process remains unclear. Proliferation of ASCs is closely related to self-renewal and FGF signaling [12].

We hypothesized that hypoxic conditions are beneficial for ASC proliferation due to self-renewal-mediated autocrine FGF–2 signaling. In the present study, ASC proliferation and the associated signaling pathways in hypoxic conditions were examined. HIF–1α expression and FGF–2 production in hypoxia were examined. A chromatin immunoprecipitation (ChIP) assay for HIF–1α binding to the hypoxia responsive element (HRE) in *FGF–2* was performed. HIF–1α was knocked down by siRNA in ASCs under hypoxia, and the mRNA expression of HIF–1α, FGF–2, and VEGF was investigated. Finally, FGF–2 and VEGF were added to ASCs, and the proliferation response was examined.

These results provide important insight into how hypoxic culture favors the ex vivo expansion of human ASCs, which will be important for maximizing the cell yield for clinical-scale ASC expansion.

## Materials and Methods

### Materials

Rabbit anti-phospho-Erk1/2, rabbit anti-phospho-Akt, rabbit anti-Akt, rabbit anti-phospho-p38, rabbit anti-p38, and rabbit anti-HIF–1α were from Epitomics Inc. (Burlingame, CA). Rabbit antibody against Erk1/2 was from Cell Signaling Technology (Beverly, MA). Rabbit antibody anti-phospho-nuclear factor kappa B (NF-ĸB) was from Abcam (Cambridge, UK). Rabbit antibodies for NF-ĸB and FGF–2 were from GeneTex Inc. (Irvine, CA). Rabbit antibody against beta-actin was from BioVision (Milpitas, CA). Mice antibody Histone H3 was Cell Signaling Technology (Beverly, MA). PD98059, an inhibitor of the MEK pathway, LY294002, an inhibitor of phosphatidylinositol-3-kinase-Akt, and SB203580, an inhibitor of p38, were from Calbiochem Novabiochem (San Diego, CA). Recombinant human VEGF and FGF–2 were purchased from PeproTech Ltd. (London, UK). Other reagents were from Sigma-Aldrich (St. Louis, MO) unless otherwise stated.

### Cell culture

The Ethics Review Board of Kansai Medical University has approved all research involving human participants and all patients provided their written consent to participate in this study. Human abdominal subcutaneous fat was collected from excess tissues excised during plastic and reconstructive surgery. ASCs were prepared as described previously [13–16]. Briefly, adipose tissue was washed extensively three times with 20 ml phosphate-buffered saline (PBS), cut into small pieces, and the extracellular matrix was digested with 0.1% collagenase solution with shaking at 37°C for 40 minutes. After adding basal medium consisting of Dulbecco’s modified Eagle’s medium (DMEM), 10% fetal bovine serum, and 1% penicillin, the cell pellet was centrifuged at 1600 rpm for 3 minutes. After removing cellular debris by filtering the cell suspension through a 100-μm nylon mesh, the cells were incubated in control medium in a dish. The adherent ASCs were maintained until passage 3 in control medium, and nearly all cells formed fibroblast-like morphology.

Hypoxic culture experiments were performed in a multigas incubator (ASTEC, Hukuoka, Japan) at 37°C in an atmosphere containing 5% CO_2_ balanced with nitrogen to reach an oxygen concentration of 1%. Normoxic culture was performed in a standard incubator in an atmosphere containing 5% CO_2_ and 20% O_2_.

### Cell proliferation assay

Cell proliferation was assessed using the commercial kits Cell Counting Kit–8 (Dojindo Molecular Technologies, Inc., Gaithersburg, MD) and DNA·IdU Labeling and Detection Kit (Takara Bio, Otsu, Japan).

The rationale for the cell counting assay using the Cell Counting Kit–8 kit is that the color-developing substrate WST–8 contained in the kit is reduced by intracellular dehydrogenase to water-soluble formazan, which can be directly quantitated photometrically. ASCs (1 × 10^4^ cells/well) were plated in 24-well plates and incubated for 1–7 days in 1% or 20% O_2_. FGF–2 (1–100 ng/mL) and VEGF (50–200 ng/mL) were added to the DMEM. The absorbance was measured at 450 nm (n = 3). The spectrometry was converted into cell number. The DNA IdU Labeling and Detection Kit is a colorimetric immunoassay based on the measurement of 5-iodo–2′-deoxyuridine (IdU) incorporation during DNA synthesis. ASCs (2 × 10^3^ cells/well) were plated in 96-well plates, incubated for 24 hours in 1% or 20% O_2_, and labeled with IdU. Cells were fixed, incubated with peroxidase-conjugated anti-IdU antibody, incubated with the peroxidase substrate 3,3′,5,5′-tetramethylbenzidine, and IdU incorporation was quantitated by measuring the optical density at 450 nm. Proliferation of ASCs in 1% O_2_ in the presence of an inhibitor (10 μM PD98059, 10 μM LY294002, or 30 μM SB203580) was examined in a similar manner.

### Western blotting

Total cell protein extracts were obtained using M-PER (Mammalian protein extraction reagent; Thermo Fisher Scientific Inc.) for the detection of phospho-Erk1/2, Erk1/2, phospho-Akt, Akt, phospho-p38, p38, phospho-NF-ĸB, NF-ĸB, and beta-actin. Nuclear protein extracts was obtained using NE-PER Nuclear and Cytoplasmic Extraction Kit (Thermo Fisher Scientific Inc., Waltham, MA) for detection of HIF–1α and Histone H3. Protein concentrations were measured using a BCA Protein assay kit (Pierce, Rockford, IL). Ten micrograms of protein extracts were separated with SDS-PAGE using a NuPAGE electrophoresis system (Invitrogen, Carlsbad, CA). Proteins were transferred to polyvinylidene difluoride membranes using the iBlot Dry Blotting System (Invitrogen) in accordance with the manufacturer’s protocol. Membrane blocking and immunodetection of proteins was performed with the WesternBreeze Chemiluminescent Detection Kit containing a secondary antibody solution of alkaline phosphatase-conjugated antibody (Invitrogen). Antibodies raised against the following proteins were used: HIF–1α (1:500), phospho-Erk1/2 (1:1000), Erk1/2 (1:1000), phospho-Akt (1:1000), Akt (1:1000), phospho-p38 (1:1000), p38 (1:1000), phospho-NF-ĸB (1:1000), NF-ĸB (1:1000), beta-actin (1:500) and Histone H3 (1:2000). We performed densitometric analysis for the western blotting result.

### RNA isolation and real-time reverse transcription-polymerase chain reaction (RT-PCR)

ASCs were plated in 60-mm cell culture dishes at 4.25× 10^5^ cells/dish. Confluent ASCs were cultured in 1% O_2_ or 20% O_2_ for 24 hours. RNA was extracted using Trizol (Life Technologies, Carlsbad, CA). Real-Time RT-PCR was performed using the One Step SYBR PrimeScript RT-PCR Kit II (TAKARA BIO INC., Otsu, Japan), according to the manufacturer’s protocol. Briefly, RT-PCR was performed in a total volume of 25 μl containing 10–100 ng total RNA, 12.5 μl 2× One Step SYBER RT-PCR Master Mix, and 1 μl PrimeScript 1-step Enzyme Mix. Each sample was analyzed in duplicate. Thermal cycler conditions were 42°C for 5 minutes and 95°C for 10 seconds, followed by 40 cycles of 95°C for 5 seconds and 60°C for 30 seconds. Amplification of the housekeeping gene *ß-actin* mRNA, which served as a normalization standard, was carried out with *ß-actin* forward (5′- TGGCACCCAGCACAATGAA -3′) and reverse (5′- CTAAGTCATAGTCCGCCTAGAAGCA -3′) primers. Side-stand-specific primers for *VEGF* and *FGF–2* were *VEGF* forward (5′-TGCTTCTGAGTTGCCCAGGA–3′) and reverse (5′-TGGTTTCAATGGTGTGAGGACATAG–3′), and *FGF–2* forward (5′- CCATCCTTTCTCCCTCGTTTCTT -3′), and reverse (5′- GATGTTTCCCTCCAATGTTTCATTC -3′).

Quantification of target cDNA (*VEGF* and *FGF–2*) and the housekeeping gene (*ß-actin*) was performed using Real-time PCR Opticon 2 (Bio-Rad Laboratories, Inc., CA, USA). Data collection and analyses were performed using the software included with the system. *VEGF* and *FGF–2* mRNA levels were measured as CT threshold levels and normalized to the individual *ß-actin* control CT values.

### ELISA for secreted growth factors

ASCs were plated in 60-mm cell culture dishes at 4.25 × 10^5^ cells/dish. Confluent ASCs were cultured in 1% O_2_ or 20% O_2_ for 24 hours. The amounts of VEGF and FGF–2 in the conditioned medium were measured with ELISA kits (R&D Systems Europe, Abingdon, UK). All experiments were performed in duplicate.

### ChIP assay

ChIP assays were performed using the EpiScope ChIP Kit (anti-mouse IgG) (Takara Bio) according to the manufacturer’s protocol. Briefly, ASCs were cultured in 1% or 20% O_2_ for 24 hours. Cells were cross-linked with 1% formaldehyde for 5 minutes, and reactions were stopped by adding Quenching solution. Then, the cells were washed, lysed, and sonicated with Bioruptor UCD–200 (COSMO BIO Co., Ltd., Tokyo, Japan) for 30 seconds 5 times in an ice bath with 60-second cooling periods between sonications to shear chromatin into smaller DNA fragments. Lysates were centrifuged, and an aliquot of supernatant was saved as input DNA. Supernatants were then immunoprecipitated with anti-HIF–1α. Immunoprecipitates were recovered by adding Magnosphere^TM^ anti-mouse IgG beads (Takara Bio). After extensive washing, 100 μl chelating resin solution was added to the beads and boiled for 15 minutes. Finally, the purified DNA was analyzed with real-time PCR for the presence of the HIF–1α-binding HRE. The PCR primers were designed to cover the HIF–1α-binding site of the human *FGF–2* fragment (nucleotides 75142 to 75264): forward, 5'-TTGGGGGAGCTGGTAACTGATG–3'; reverse, 5'-CAGTAGATGTTTCCCTCCAATG–3'. Ten percent of the lysate was used as the input control for PCR. The ChIP-precipitated DNA and input DNA were subjected to real-time PCR analyses using the One Step SYBR PrimeScript RT-PCR Kit II (TAKARA BIO), and samples from two individual ChIP assays were analyzed in triplicate. The results were normalized to the input and expressed as the n-fold increase over those of the normoxic controls.

### siRNA transfection

One day before transfection, 25,000 cells of ASCs were plated in 2500 μl of growth medium without antibiotics in a 6-well plate. The cell density was 30–50% confluent at the time of transfection. For each well to be transfected, RNAi duplex-Lipofectamine™ RNAiMAX complexes were prepared as follows: 150 pmol RNAi (10 μl, esiRNA human HIF1A, Sigma-Aldrich, St. Louis, MO, USA) was diluted in 1250 μl of Opti-MEM® I Reduced Serum Medium without serum (Invitrogen, Carlsbad, CA, USA) gently. Lipofectamine™ RNAiMAX (25 μl) was diluted in Opti-MEM® I Reduced Serum Medium (1250 μl) gently. RNAi duplex with the diluted Lipofectamine™ RNAiMAX was combined and incubated for 10–20 minutes at room temperature. RNAi duplex-Lipofectamine™ RNAiMAX complexes (500 μl) were added to each well containing cells. This gave a final volume of 3,000 μl and a final RNA concentration of 10 nM. The cells were incubated for 48 hours at 37°C in a CO_2_ incubator until the time of the assay for gene knockdown. Knockdown was evaluated by real-time RT-PCR of HIF–1α and FGF–2. The real-time PCR method and primers of FGF–2 and the housekeeping β-actin gene were the same as those for the above-mentioned RT-PCR described in Materials & Methods. Strand-specific primers were as follows: HIF–1α forward (5’-TTGCTCATCAGTTGCCACTTCC–3’) and reverse (5’-AGCAATTCATCTGTGCTTTCATGTC–3’). The universal negative control (Nippon Gene, Co., Ltd., Tokyo) was used as siRNA control in the experiments.

### Statistical analysis

The Mann-Whitney U test was used for comparisons between groups, with p < 0.05 considered significant. Data are the means ± SD.

## Results

### Hypoxia promotes proliferation of ASCs

**Fig 1:**
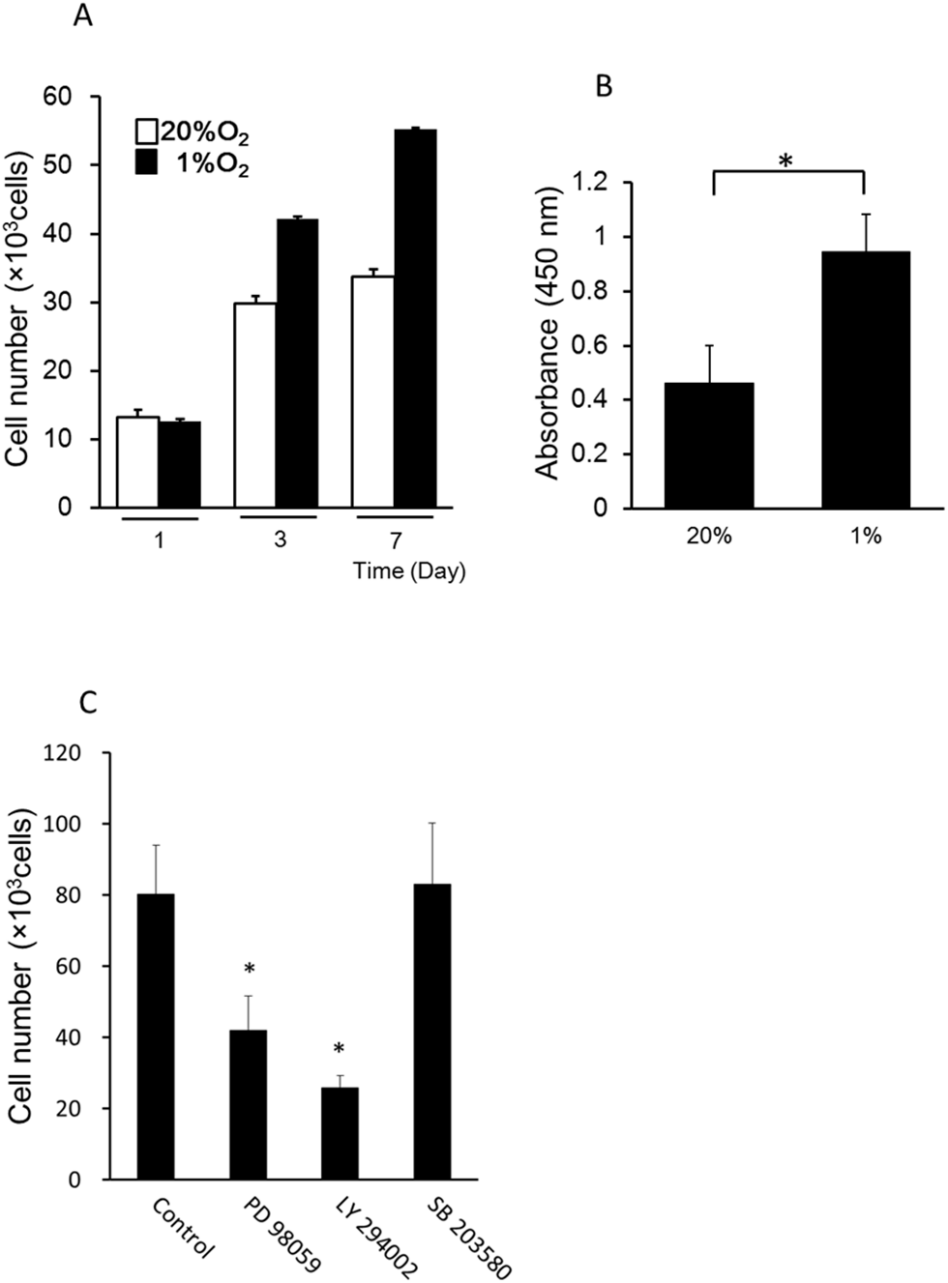
(A) Effects of hypoxia on the proliferation of human adipose-derived stem cells (ASCs). Proliferation was measured using Cell Counting Kit–8 according to the manufacturer’s instructions. A significant difference in ASC proliferation was observed between the hypoxia and normoxia groups on days 3 and 7. (B) Effects of hypoxia on the proliferation of human ASCs. DNA synthetic quantity was measured using the DNA IdU Labeling and Detection Kit according to the manufacturer’s instructions. A significant difference in DNA synthetic quantity of ASC was observed between the hypoxia and normoxia groups. (C) Effects of PD98059, LY294002, and SB203580 on the proliferation of human ASCs in hypoxia. Cell proliferation during hypoxia was significantly suppressed by PD98059 (10 μM) and LY294002 (10 μM) but not SB203580. Data are the means ± SD. *p < 0.05 vs. control.

We first examined the effect of hypoxia on ASC proliferation. As shown in Fig 1A, the number of ASCs in 1% O_2_ was higher than that in 20% O_2_ using Cell Counting Kit–8. Compared with normoxia, ASCs cultured in 1% O_2_ showed 1.5-fold higher proliferation on day 7. As shown in Fig 1B, in DNA synthetic quantity of hypoxia ASC was increased significantly compared with normoxia. Cell proliferation in hypoxia was significantly suppressed by PD98059 and LY294002 but not SB203580 (Fig 1C).

### Hypoxia induces Erk and Akt phosphorylation and induces HIF–1α expression in ASCs

**Fig 2:**
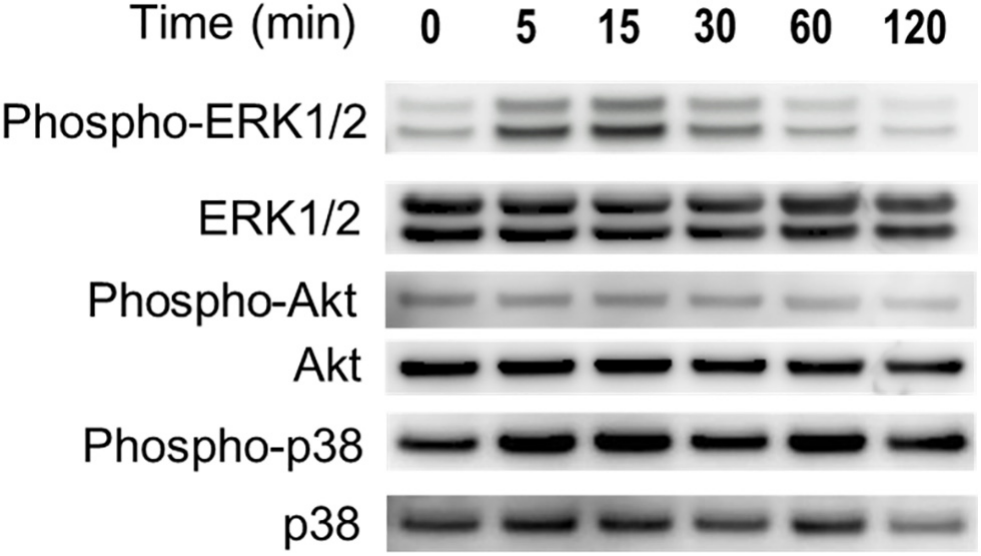
Effects of hypoxia on the expression of Erk, Akt, p38, and NF-ĸB in ASCs. Hypoxia activated the Erk and Akt pathways. Cell lysates were prepared from ASCs exposed to hypoxia for the indicated times, and the phosphorylation levels of ERK1/2, Akt, p38 and were determined with western blotting.

**Fig 3:**
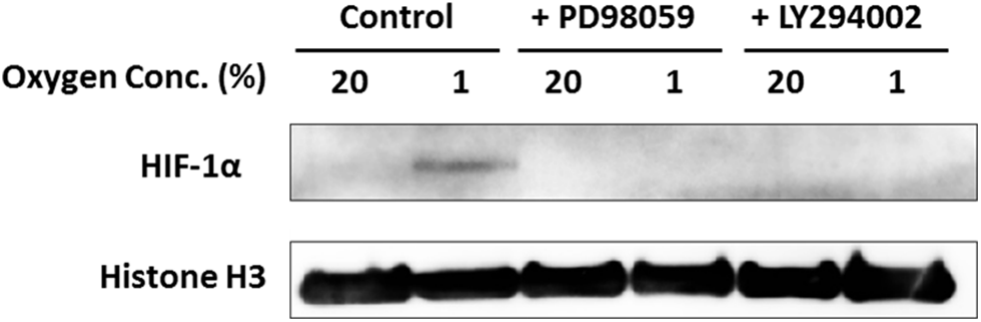
Effects of hypoxia on the expression of HIF–1α in ASCs. Nuclear protein was prepared from ASCs exposed to hypoxia for 6 hours, and western blotting was performed. HIF–1α was increased in hypoxia. However, the expression of HIF–1α of ASCs in hypoxia with inhibitor (PD98059: 10 μM; LY294002: 20 μM) was not detected by western blotting.

We investigated the activation of Erk1/2, Akt, p38, and NF-ĸB in ASCs in hypoxia. Phosphorylation of Erk1/2 and Akt was mostly seen after 5–15 minutes in hypoxia. Phosphorylation of p38 (Fig 2). Phosphorylation of NF-ĸB was not detected in hypoxia (data not shown). In the densitometric analysis, the intensity of the phospho-ERK1/2 and phospho-Akt protein signal increased significantly with under hypoxia as sown in S1 Fig. Therefore, we concluded that only phospho-ERK 1/2 and Akt were responsible for ASC proliferation. We also evaluated the amount of HIF–1α protein to demonstrate that the cells were actually exposed to low oxygen. HIF–1α protein was increased in hypoxia. In addition, we examined whether Erk and Akt inhibitors suppressed the HIF–1α expression. The expression of HIF–1α of ASCs in 1% O_2_ with inhibitor (PD98059: 10 μM; LY294002: 20 μM) was not detected by western blotting, as shown in Fig 3.

### Hypoxia induces the mRNA expression of *VEGF* and *FGF–2*

**Fig 4:**
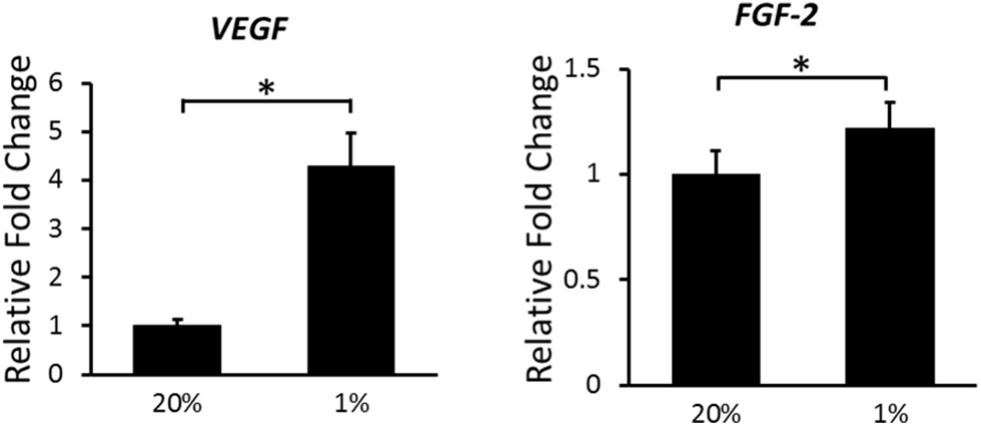
Effects of hypoxia on mRNA expression of *VEGF* and *FGF–2* in ASCs. After incubation for 24 hours, the mRNA expression of the indicated genes was analyzed using real-time RT-PCR. The fold-change during hypoxia is expressed relative to the level in normoxia. *VEGF* and *FGF–2* expression in hypoxia was significantly higher than in normoxia. Data are the means ± SD. *p < 0.05.

To determine the induction of expression of hypoxia-associated growth factor genes, we measured mRNA levels of *VEGF* and *FGF–2* with real-time RT-PCR. The *VEGF* and *FGF–2* expression levels were significantly increased 4.28-fold and 1.21-fold, respectively, in hypoxia compared to normoxia at 24 hours (Fig 4).

### Hypoxia induces the secretion of VEGF and FGF–2

**Fig 5:**
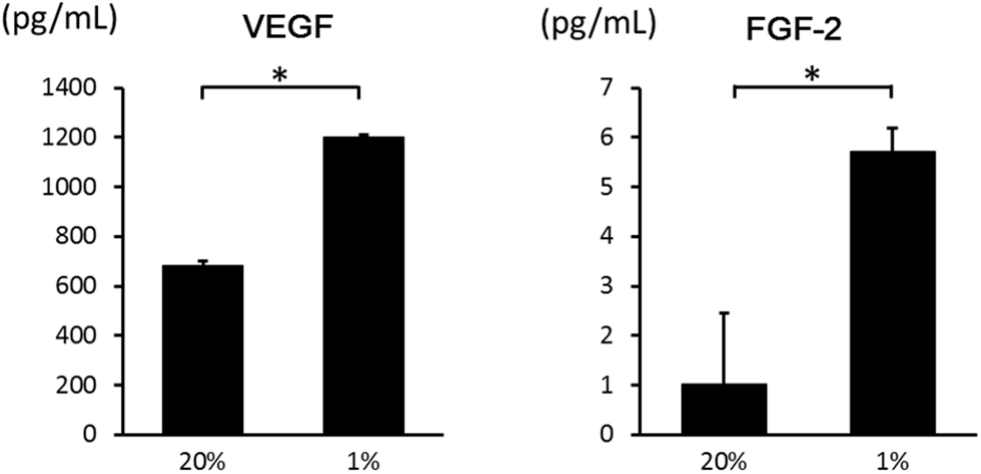
Effects of hypoxia on the secretion of VEGF and FGF–2 from ASCs. The secretion of VEGF and FGF–2 into the medium in hypoxia was significantly higher than in normoxia. Data are the means ± SD. *p < 0.05.

**Fig 6:**
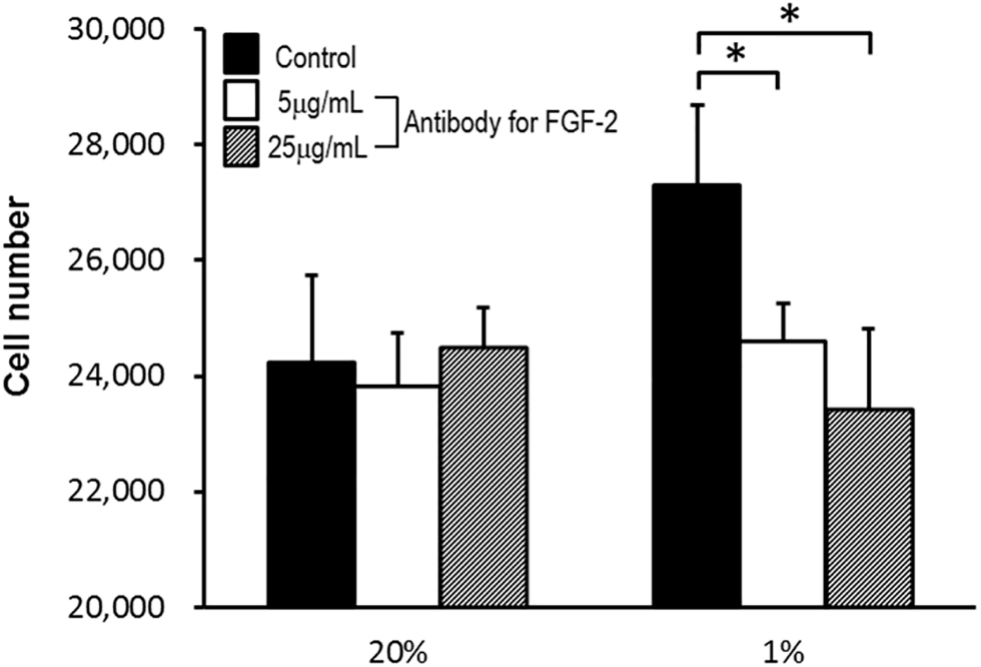
Effects of anti FGF–2 antibodies on proliferation of ASCs. ASCs were cultured in media with 0 (control), 5 and 25 μg/mL of the antibodies under 20% O_2_ and 1% O_2_ conditions for 48h. Proliferation was measured using Cell Counting Kit–8 according to the manufacturer’s instructions and the absorbance was converted into cell number. The proliferation of ASCs under 1% O_2_ was inhibited by the antibodies. Data are the means ± SD. *p < 0.01.

Secreted growth factors in the medium were measured with ELISA in hypoxia and normoxia at 24 hours. Protein levels of VEGF and FGF–2 were significantly increased in hypoxia (Fig 5). Therefore, hypoxia promoted the secretion of both VEGF and FGF–2. When culture media under hypoxia was treated with antibody for FGF–2, ASC proliferation almost diminished (Fig 6). These results confirm that FGF–2 promotes ASC proliferation. The results above were added in the text.

### FGF–2 induces ASC proliferation

**Fig 7:**
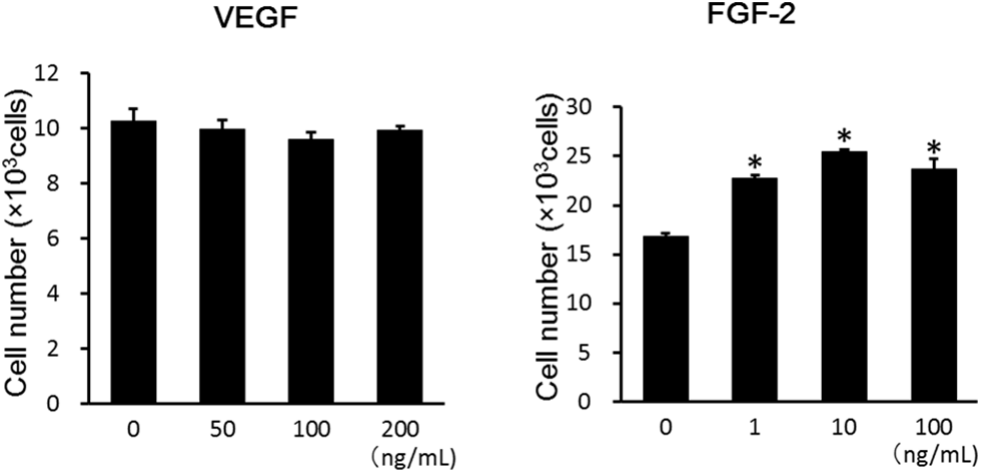
Effects of VEGF and FGF–2 on proliferation of ASCs. Proliferation was measured using Cell Counting Kit–8 1 day after addition growth factors according to the manufacturer’s instructions. Compared with the control group that was not treated with VEGF and FGF–2, no significant difference in ASC proliferation was observed in groups treated with VEGF. In contrast, FGF–2 significantly promoted cell proliferation in a dose-dependent manner.

VEGF and FGF–2 were added to the medium to examine their effects on proliferation of ASCs. Compared with the control group that was not treated with VEGF and FGF–2, no significant difference in ASC proliferation was observed in groups treated with VEGF. In contrast, FGF–2 significantly promoted cell proliferation in a dose-dependent manner (Fig 7).

### Functional HIF–1αbinding to the HRE in *FGF–2* in ASCs

**Fig 8:**
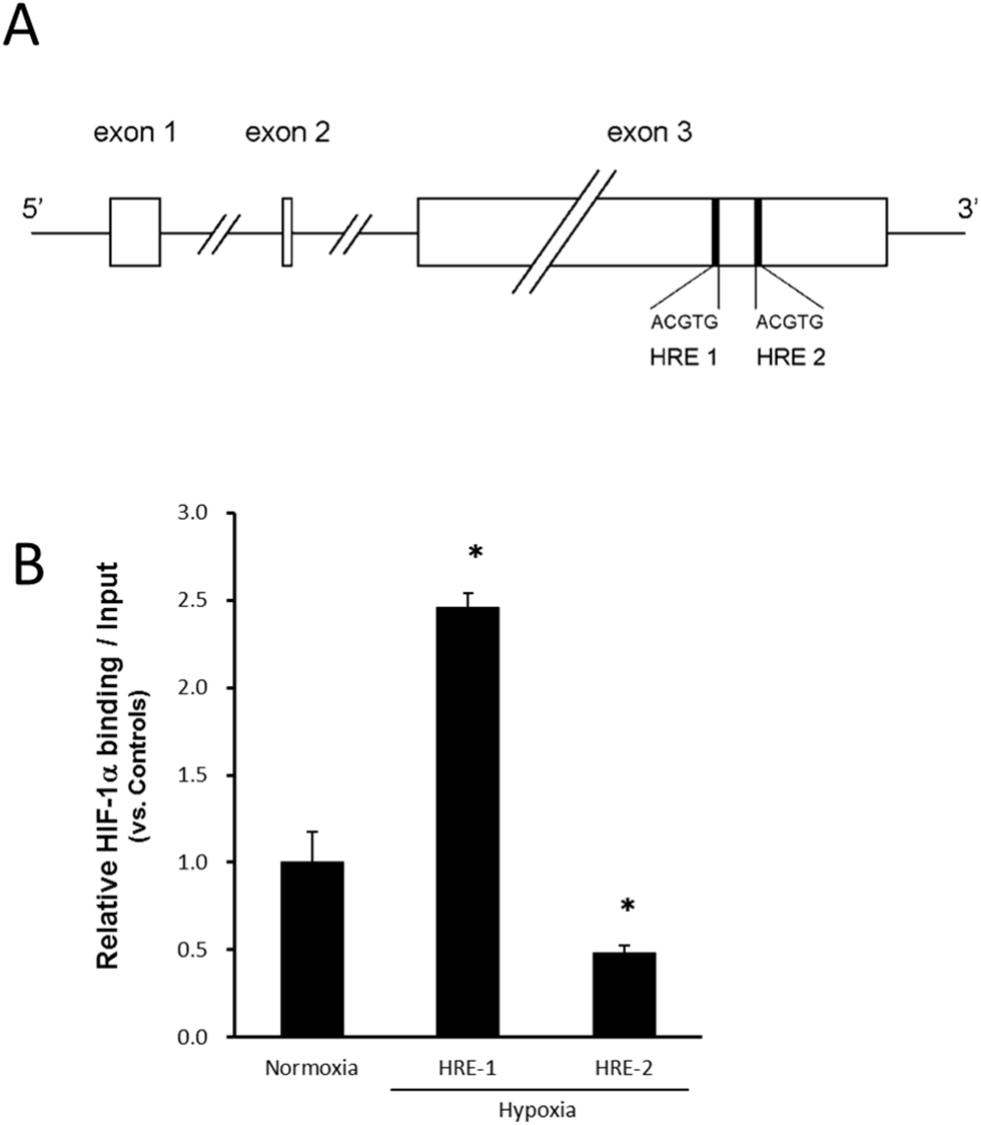
(A) The HIF–1α-binding responsive element (5'-ACGTG–3') is called the HRE. HRE was present at 2 sites in the FGF–2 gene, and these were designated as HRE1 and HRE2. (B) Functional HIF–1α binding to hypoxia responsive element (HRE) found in the proximal region of *FGF–2* in hypoxia. The relative association of HIF–1α with human *FGF–2* was analyzed with ChIP in ASCs incubated in normoxia (20% O_2_) or hypoxia (1% O_2_) for 24 hours. Sheared chromatin was immunoprecipitated with anti-HIF–1α. The enrichment of HIF–1α was quantified with real-time PCR using HRE-specific primers for *FGF–2*. In response to hypoxia, the binding for HRE 1 was significantly enhanced about 2.5-fold. Data were normalized to the total amount of added DNA and are the means ± SD of two independent experiments performed in triplicate. *p < 0.05 vs. normoxia.

The HIF–1α-binding responsive element (5'-ACGTG–3') called the HRE in *FGF–2* is shown in Fig 8A. HRE was present at 2 sites (HRE1 and HRE2) in exon 3 of the FGF–2 gene. To clarify the interaction of HIF–1α with one of its target genes in ASCs, we examined HIF–1α binding to *FGF–2* and the promoter. The protein-DNA interaction was examined at the HRE found in *FGF–2* in ASCs using the ChIP assay. The ChIP assay of HIF–1α was performed for each of HRE1 and HRE2, but amplification by PCR was confirmed at only one site (HRE1). In response to hypoxia, the binding for HRE1 was enhanced about 2.5-fold (Fig 8B). In contrast, the ChIP assay for HIF–1α in the *FGF–2* promoter revealed that HIF–1α did not bind to the HRE site (data not shown).

### HIF–1α knockdown significantly decreases the expression of FGF–2 on ASCs

**Fig 9:**
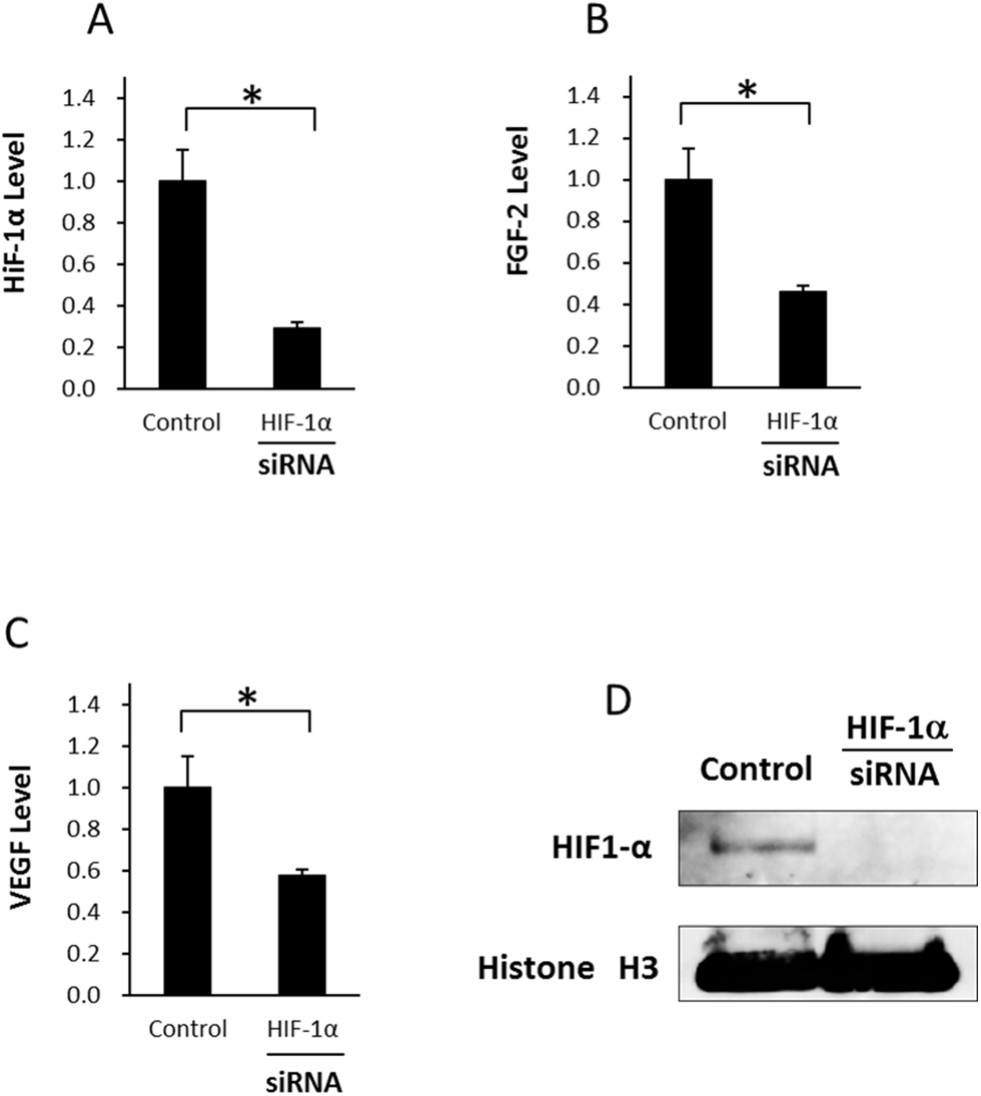
Influence of HIF–1α knockdown by siRNA in ASCs under hypoxia (A) The relative mRNA expression levels of HIF–1α in ASCs after transfection for 48 h under hypoxia. HIF–1α level was suppressed significantly compared with that in the control. (B) The relative mRNA expression levels of FGF–2 in ASCs after transfection for 48 h under hypoxia. FGF–2 level was suppressed significantly compared with that in the control. (C) The relative mRNA expression levels of VEGF in ASCs after transfection for 48 h under hypoxia. VEGF level was suppressed significantly compared with that in the control. Data are the means ± SD. *p < 0.05. (D) Expression of HIF–1α was inhibited by siRNA using western blotting.

For further determination of the effect of HIF–1α knockdown in hypoxia, we measured the expression levels of HIF–1α, FGF–2, and VEGF (Fig 9A, 9B and 9C). Transfection with HIF–1α siRNA in hypoxic culture inhibited protein expression of HIF–1α in western blotting. Transfection with HIF–1α siRNA in hypoxic culture inhibited HIF–1α mRNA expression by about 70%, and the levels of FGF–2 and VEGF were inhibited by about 54% and 43%, respectively, showing that HIF–1α expression under hypoxia is closely related to FGF–2 and VEGF expressions. Furthermore, we showed the inhibition level of HIF–1α by siRNA using western blotting (Fig 9D).

## Discussion

This study firstly demonstrated that hypoxia enhanced the proliferation of ASCs via HIF–1α activation. The hypoxic culture stimulated the phosphorylation of ERK1/2 and Akt-induced HIF–1α expression. The levels of *VEGF* and *FGF–2* mRNA and their proteins in the ASCs were significantly enhanced in the hypoxic condition. Of these growth factors, FGF–2 affected ASC proliferation. The ChIP assay revealed that the binding of HIF–1α to *FGF–2* increased in hypoxia. HIF–1α activation, FGF–2 production, and the ERK1/2 and Akt pathway were involved in this regulatory mechanism.

ASCs were recently isolated from human subcutaneous adipose tissue [1] [13] [14]. ASCs may be useful in regenerative medicine as an alternative to BMCs. Changes in the oxygen concentration influence physiological activities, such as stem cell proliferation. For example, Lennon et al. showed that culturing BMCs from 6- to 12-week-old rats in 5% pO_2_ resulted in an approximately 40% higher cell number at the first passage compared with culturing these cells in 21% pO_2_ [17]. Grayson et al. also reported that human BMCs show enhanced proliferation in hypoxia (2% pO_2_) over seven passages, resulting in a 30-fold increase in cell number compared with normoxia [7]. However, the effects of the oxygen concentration on ASCs remain unclear. In the present study, we found that the proliferation-inducing effects of hypoxia on ASCs were similar to those on BMCs.

Several researchers have reported that ASC proliferation is more markedly influenced under hypoxic versus normoxic conditions in vitro. Lee et al. reported that the proliferation of human ASCs is significantly increased in hypoxia (2%) [11]. Yamamoto et al. also reported that ASCs cultured in 2% O_2_ show a 1.5-fold increase in proliferation over 6 weeks of culture [8]. In addition, Rasmussen compared the effects of prolonged hypoxic culture on growth of human ASCs cultured in 1, 5, and 21% oxygen. They concluded that culturing ASCs in 5% oxygen significantly lowers the ASC doubling time among the groups [18]. Using the IdU incorporation assay and cell number counting assay, we demonstrated that ASC proliferation in hypoxia (1%) was increased compared with normoxia. An oxygen tension of 1–5% in culture is reported to be sufficient to increase the proliferation of ASCs. Collectively, these results support the idea that hypoxic culture conditions are favorable for ASCs. Hypoxic culture allows the production of many ASCs from a few donor cells, providing a useful culture method for the large-scale production of ASCs that will be required in regenerative medicine.

HIF is induced by insufficient oxygen supply to cells and functions as a transcription factor. HIF-α (HIF–1α, -2α, and -3α) binds to constitutively expressed HIF–1β in cells, forming a heterodimer. HIF–1α is also produced under normoxic conditions, but does not function because it is degraded by the 26S proteasome, a proteolytic enzyme complex. HIF–1α is unlikely to be degraded in hypoxia, and it migrates into the nucleus to form a heterodimer with HIF–1β or binds to histone acetylation enzymes, such as CBP (CREB1-binding protein) /p300 [19]. The resulting complexes bind to a response element called the HRE (5'-ACGTG–3'). Asp803 of HIF–1α is involved in the transport of the molecular complex CBP/p300 with histone acetyltransferase activity to HRE on DNA, promoting transcription of various genes. HIF–1α induces the expression of VEGF, platelet-derived growth factor, and basic fibroblast growth factor, enhancing angiogenesis and eventually improving the oxygen environment [20]. However, the roles of HIF–1α in ASCs remain unclear.

The expression of HIF–1α in ASCs during hypoxia has been reported by several researchers [8] [21]. Stubbs et al. reported that HIF–1α becomes stabilized during hypoxia due to an increase in VEGF-A protein secretion [21]. Yamamoto et al. also reported that HIF–1α protein is increased in hypoxic conditions due to significantly enhanced VEGF secretion [8]. Lee et al. demonstrated that expression of *VEGF* and *FGF–2* mRNA is enhanced in ASCs in hypoxia, promoting the secretion of these growth factors [11]. However, the relationship between the mRNA and protein expression of FGF–2 and the expression of HIF–1α in hypoxia was unclear. In the present study, we first demonstrated that HIF–1α binds to an HRE in FGF–2 gene in ASCs to enhance mRNA and protein expression of FGF–2, thereby promoting proliferation.

Akt and p38 phosphorylation lead to the stabilization of HIF–1α and the survival response in BMCs [22]. HIF–1α stabilization leads to the induction of HIF–1α-responsive genes including proangiogenic factors such as VEGF and interleukin–6 [23]. In the present study, we demonstrated that Akt was phosphorylated in the hypoxic culture of ASCs. Activated Akt may stabilize HIF–1α as a survival response in ASCs, as is seen in BMCs. Kim et al. reported that the expression levels of phosphorylated ERK1/2 and Akt are increased as well as the proliferation of ASCs in hypoxia [24]. Our results also demonstrated that ERK1/2 and Akt were phosphorylated along with the proliferation of ASCs in hypoxia.

FGF–2 plays a critical autocrine/paracrine role in human ASC self-renewal [12]. The effects of VEGF on ASCs vary in different published reports. Suga et al. reported no significant effect after the addition of VEGF to the medium of ASCs [25]. In contrast, VEGF treatment significantly increased bromodeoxyuridine incorporation, indicating increased proliferation of ASCs. In this study, VEGF did not affect the cell proliferation of ASCs, although FGF–2 promoted proliferation. Thus, of the growth factors produced by ASCs in hypoxia, FGF–2 was involved in cell proliferation.

The relationship between ASC proliferation and differentiation and reactive oxygen species (ROS) has recently been attracting attention [26], and a role for ROS generation as a key mediator of ASC proliferation under hypoxia has been proposed [24] [27] [28]. Further investigation is necessary to clarify the relationship between ASC proliferation under hypoxia and ROS.

In conclusion, the mRNA and protein expression of FGF–2 was enhanced as well as the proliferation of ASCs in hypoxia. HIF–1α expression and ERK1/2 and Akt phosphorylation were observed in hypoxia. HRE, a binding site fir HIF–1α, was present in *FGF–2* in ASCs. These results demonstrate that HIF–1α expression is strongly involved in cell proliferation in hypoxia, revealing a component of the kinetics and cell regulatory mechanisms of ASCs in hypoxia. Hypoxic culture may be a convenient method to enhance the proliferative capacity of stem cells for transplantation. Although the effects of hypoxic culture on ASCs should be further investigated, ASCs cultured on a large scale in hypoxia using this method of controlling proliferation may be clinically useful.

## Supporting Information

S1 Fig The quantities of phospho-ERK1/2, ERK1/2, Phospho-Akt, Akt, phospho-p38 and p38 were determined using densitometry. Data are the means ± SD of 4 independent experiments. The expression levels of phospho-ERK1/2 were normalized to ERK1/2 levels in the same sample. *p < 0.05 compared with 0 hours. The expression of Akt and p38 was analyzed in the same manner. The intensity of the phospho-ERK1/2 and phospho-Akt protein signal increased significantly with under hypoxia.
(TIF)

## References

[1] Zuk PA, Zhu M, Ashjian P, De Ugarte DA, Huang JI, Mizuno H, et al Human adipose tissue is a source of multipotent stem cells. Molecular biology of the cell, 2002 13: 4279–4295. 12475952 10.1091/mbc.E02-02-0105 PMC138633

[2] Mizuno H, Tobita M, Uysal AC. Concise review: Adipose-derived stem cells as a novel tool for future regenerative medicine. Stem Cells, 2012 30: 804–810. 10.1002/stem.1076 22415904

[3] Rajala K, Lindroos B, Hussein SM, Lappalainen RS, Pekkanen-Mattila M, Inzunza J, et al A defined and xeno-free culture method enabling the establishment of clinical-grade human embryonic, induced pluripotent and adipose stem cells. PloS one, 2010 5: e10246 10.1371/journal.pone.0010246 20419109 PMC2856688

[4] Zeng G, Lai K, Li J, Zou Y, Huang H, Liang J, et al A rapid and efficient method for primary culture of human adipose-derived stem cells. Organogenesis, 2013 9: 287–295. 10.4161/org.27153 24280895 PMC3903698

[5] Ezashi T, Das P, Roberts RM. Low O_2_ tensions and the prevention of differentiation of hES cells. Proceedings of the National Academy of Sciences of the United States of America, 2005 102: 4783–4788. 15772165 10.1073/pnas.0501283102 PMC554750

[6] Yoshida Y, Takahashi K, Okita K, Ichisaka T, Yamanaka S. Hypoxia enhances the generation of induced pluripotent stem cells. Cell Stem Cell, 2009 5: 237–241. 10.1016/j.stem.2009.08.001 19716359

[7] Grayson WL, Zhao F, Bunnell B, Ma T. Hypoxia enhances proliferation and tissue formation of human mesenchymal stem cells. Biochem Biophys Res Commun, 2007 358: 948–953. 17521616 10.1016/j.bbrc.2007.05.054

[8] Yamamoto Y, Fujita M, Tanaka Y, Kojima I, Kanatani Y, Ishihara M, et al Low oxygen tension enhances proliferation and maintains stemness of adipose tissue-derived stromal cells. Biores Open Access, 2013 2: 199–205. 10.1089/biores.2013.0004 23741631 PMC3666216

[9] Hung SP, Ho JH, Shih YR, Lo T, Lee OK. Hypoxia promotes proliferation and osteogenic differentiation potentials of human mesenchymal stem cells. J Orthop Res, 2012 30: 260–266. 10.1002/jor.21517 21809383

[10] Dos Santos F, Andrade PZ, Boura JS, Abecasis MM, da Silva CL, Cabral JMS. Ex vivo expansion of human mesenchymal stem cells: a more effective cell proliferation kinetics and metabolism under hypoxia. Journal of cellular physiology, 2010 223: 27–35. 10.1002/jcp.21987 20020504

[11] Lee EY, Xia Y, Kim WS, Kim MH, Kim TH, Kim KJ, et al Hypoxia-enhanced wound-healing function of adipose-derived stem cells: increase in stem cell proliferation and up-regulation of VEGF and bFGF. Wound repair and regeneration: official publication of the Wound Healing Society [and] the European Tissue Repair Society, 2009 17: 540–547. 10.1111/j.1524-475X.2009.00499.x 19614919

[12] Zaragosi LE, Ailhaud G, Dani C. Autocrine fibroblast growth factor 2 signaling is critical for self-renewal of human multipotent adipose-derived stem cells. Stem Cells, 2006 24: 2412–2419. 16840552 10.1634/stemcells.2006-0006

[13] Kakudo N, Shimotsuma A, Miyake S, Kushida S, Kusumoto K. Bone tissue engineering using human adipose-derived stem cells and honeycomb collagen scaffold. J Biomed Mater Res A, 2008 84: 191–197. 17607760 10.1002/jbm.a.31311

[14] Kakudo N, Shimotsuma A, Kusumoto K. Fibroblast growth factor–2 stimulates adipogenic differentiation of human adipose-derived stem cells. Biochem Biophys Res Commun, 2007 359: 239–244. 17543283 10.1016/j.bbrc.2007.05.070

[15] Kakudo N, Minakata T, Mitsui T, Kushida S, Notodihardjo FZ, Kusumoto K. Proliferation-promoting effect of platelet-rich plasma on human adipose-derived stem cells and human dermal fibroblasts. Plastic and reconstructive surgery, 2008 122: 1352–1360. 10.1097/PRS.0b013e3181882046 18971718

[16] Kakudo N, Kushida S, Suzuki K, Matsumoto N, Kusumoto K. Effect of C3 transferase on human adipose-derived stem cells. Hum Cell, 2011 24: 165–169. 10.1007/s13577-011-0033-0 21984005

[17] Lennon DP, Edmison JM, Caplan AI. Cultivation of rat marrow-derived mesenchymal stem cells in reduced oxygen tension: effects on in vitro and in vivo osteochondrogenesis. Journal of cellular physiology, 2001 187: 345–355. 11319758 10.1002/jcp.1081

[18] Rasmussen JG, Frobert O, Pilgaard L, Kastrup J, Simonsen U, Zachar V, et al Prolonged hypoxic culture and trypsinization increase the pro-angiogenic potential of human adipose tissue-derived stem cells. Cytotherapy, 2011 13: 318–328. 10.3109/14653249.2010.506505 20795759

[19] Yamashita K, Discher DJ, Hu J, Bishopric NH, Webster KA. Molecular regulation of the endothelin–1 gene by hypoxia. Contributions of hypoxia-inducible factor–1, activator protein–1, GATA–2, AND p300/CBP. The Journal of biological chemistry, 2001 276: 12645–12653. 11278891 10.1074/jbc.M011344200

[20] Rey S, Semenza GL. Hypoxia-inducible factor-1-dependent mechanisms of vascularization and vascular remodelling. Cardiovascular research, 2010 86: 236–242. 10.1093/cvr/cvq045 20164116 PMC2856192

[21] Stubbs SL, Hsiao ST, Peshavariya HM, Lim SY, Dusting GJ, Dilley RJ. Hypoxic preconditioning enhances survival of human adipose-derived stem cells and conditions endothelial cells in vitro. Stem cells and development, 2012 21: 1887–1896. 10.1089/scd.2011.0289 22165914

[22] Kanichai M, Ferguson D, Prendergast PJ, Campbell VA. Hypoxia promotes chondrogenesis in rat mesenchymal stem cells: a role for AKT and hypoxia-inducible factor (HIF)-1alpha. Journal of cellular physiology, 2008 216: 708–715. 10.1002/jcp.21446 18366089

[23] Hu X, Yu SP, Fraser JL, Lu Z, Ogle ME, Wang JA, et al Transplantation of hypoxia-preconditioned mesenchymal stem cells improves infarcted heart function via enhanced survival of implanted cells and angiogenesis. The Journal of thoracic and cardiovascular surgery, 2008 135: 799–808. 10.1016/j.jtcvs.2007.07.071 18374759

[24] Kim JH, Park SH, Park SG, Choi JS, Xia Y, Sung JH. The pivotal role of reactive oxygen species generation in the hypoxia-induced stimulation of adipose-derived stem cells. Stem cells and development, 2011 20: 1753–1761. 10.1089/scd.2010.0469 21265612 PMC3182032

[25] Suga H, Eto H, Shigeura T, Inoue K, Aoi N, Kato H, et al IFATS collection: Fibroblast growth factor-2-induced hepatocyte growth factor secretion by adipose-derived stromal cells inhibits postinjury fibrogenesis through a c-Jun N-terminal kinase-dependent mechanism. Stem Cells, 2009 27: 238–249. 10.1634/stemcells.2008-0261 18772314

[26] Kim JH, Kim SH, Song SY, Kim WS, Song SU, Yi TG, et al Hypoxia induces adipocyte differentiation of adipose-derived stem cells by triggering reactive oxygen species generation. Cell biology international, 2014 38: 32–40. 10.1002/cbin.10170 23956071

[27] Kim JH, Song SY, Park SG, Song SU, Xia Y, Sung JH et al Primary involvement of NADPH oxidase 4 in hypoxia-induced generation of reactive oxygen species in adipose-derived stem cells. Stem cells and development, 2012 21: 2212–2221. 10.1089/scd.2011.0561 22181007 PMC3411360

[28] Kang S, Kim SM, Sung JH. Cellular and molecular stimulation of adipose-derived stem cells under hypoxia. Cell biology international, 2014 38: 553–562. 10.1002/cbin.10246 24446066

