# Supplementary figures and images for: Hypoxia Enhances Proliferation of Human Adipose-Derived Stem Cells via HIF-1ɑ Activation
Source: PLoS One. 2015 Oct 14;10(10):e0139890. doi: 10.1371/journal.pone.0139890 (PMC4605777; doi:10.1371/journal.pone.0139890)

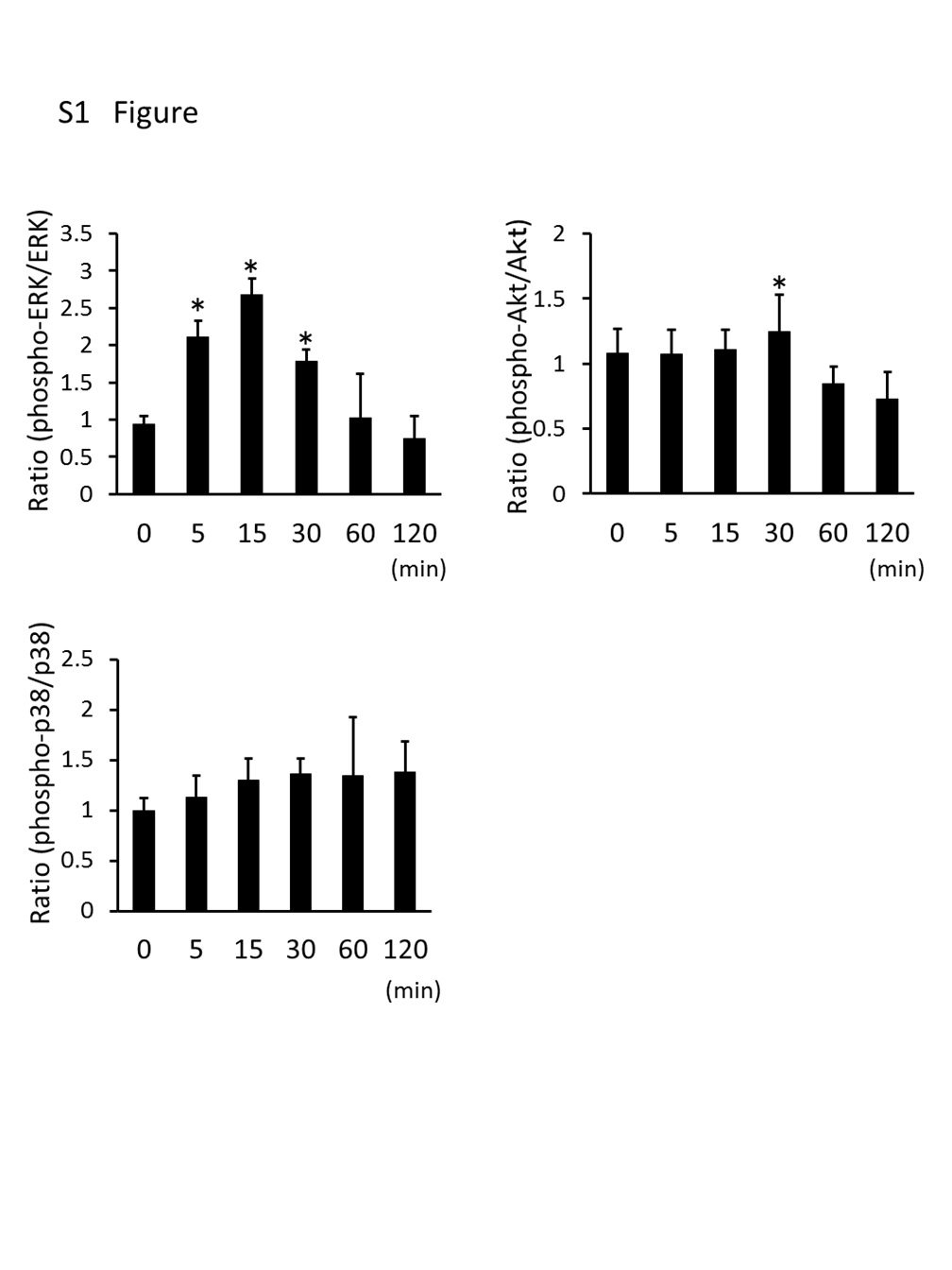

Supplement: S1 Fig — Data are the means ± SD of 4 independent experiments. The expression levels of phospho-ERK1/2 were normalized to ERK1/2 levels in the same sample. *p < 0.05 compared with 0 hours. The expression of Akt and p38 was analyzed in the same manner. The intensity of the phospho-ERK1/2 and phospho-Akt protein signal increased significantly with under hypoxia. (TIF) [file pone.0139890.s001.tif]
